# *Lactobacillus* (LA-1) and butyrate inhibit osteoarthritis by controlling autophagy and inflammatory cell death of chondrocytes

**DOI:** 10.3389/fimmu.2022.930511

**Published:** 2022-10-17

**Authors:** Keun-Hyung Cho, Hyun Sik Na, JooYeon Jhun, Jin Seok Woo, A Ram Lee, Seung Yoon Lee, Jeong Su Lee, In Gyu Um, Seok Jung Kim, Sung-Hwan Park, Mi-La Cho

**Affiliations:** ^1^ Rheumatism Research Center, Catholic Research Institute of Medical Science, Catholic University of Korea, Seoul, South Korea; ^2^ Department of Biomedicine & Health Sciences, College of Medicine, Catholic University of Korea, Seoul, South Korea; ^3^ Department of Orthopedic Surgery, College of Medicine, Catholic University of Korea, Seoul, South Korea; ^4^ Division of Rheumatology, Department of Internal Medicine, Seoul St. Mary’s Hospital, College of Medicine, Catholic University of Korea, Seoul, South Korea; ^5^ Department of Medical Life Sciences, College of Medicine, Catholic University of Korea, Seoul, South Korea

**Keywords:** osteoarthritis, inflammation, microbiota, LA-1, butyrate, necroptosis

## Abstract

Osteoarthritis (OA) reduces the quality of life as a result of the pain caused by continuous joint destruction. Inactivated *Lactobacillus* (LA-1) ameliorated osteoarthritis and protected cartilage by modulating inflammation. In this study, we evaluated the mechanism by which live LA-1 ameliorated OA. To investigate the effect of live LA-1 on OA progression, we administered LA-1 into monosodium iodoacetate (MIA)-induced OA animals. The pain threshold, cartilage damage, and inflammation of the joint synovial membrane were improved by live LA-1. Furthermore, the analysis of intestinal tissues and feces in the disease model has been shown to affect the systems of the intestinal system and improve the microbiome environment. Interestingly, inflammation of the intestinal tissue was reduced, and the intestinal microbiome was altered by live LA-1. Live LA-1 administration led to an increase in the level of *Faecalibacterium* which is a short-chain fatty acid (SCFA) butyrate-producing bacteria. The daily supply of butyrate, a bacterial SCFA, showed a tendency to decrease necroptosis, a type of abnormal cell death, by inducing autophagy and reversing impaired autophagy by the inflammatory environment. These results suggest that OA is modulated by changes in the gut microbiome, suggesting that activation of autophagy can reduce aberrant cell death. In summary, live LA-1 or butyrate ameliorates OA progression by modulating the gut environment and autophagic flux. Our findings suggest the regulation of the gut microenvironment as a therapeutic target for OA.

## Introduction

Osteoarthritis (OA) is the most common joint disorder, principally of the diarthrodial joints, and has an increasing socioeconomic effect due to aging of the population (1–4). The severe pain and irreversible cartilage destruction connected with OA reduce the quality of life (3–6). Osteoarthritis has multiple causes, including obesity, metabolic syndrome, age, genetics, gender, joint injury, and gut microbiota (7–11).

There is a relationship between the microbiome and OA (9, 10, 12). OA animal models and patients showed a relationship between an increased level of the inflammatory marker, bacterially derived lipopolysaccharide (LPS), and OA disease activity, implicating microbiome-derived proinflammatory metabolites in OA (13, 14). Schott et al. showed that oligofructose supplement ameliorates OA progression in high-fat diet-fed rodents by reducing inflammation in the colon and knee (15). The abundance of streptococcus species is reportedly linked to extended knee pain in patients with OA (12). Huang et al. reported that transplantation of fecal matter of OA patients with metabolic syndrome to germ-free mice enhanced gut permeability and systemic low-grade inflammation and aggravated disease severity in an OA mouse model (16). These results suggest that manipulation of the gut microbiome has therapeutic potential for OA.

The development and progression of OA are related to apoptosis, oxidative stress, senescence, autophagy, and necroptosis of chondrocytes (17, 18). The mammalian target of rapamycin (mTOR) is a major repressor of autophagy, a cell survival mechanism. Inhibition of cartilage-specific mTOR enhances autophagy and decreases disease activity in experimental OA models (19, 20). Also, cartilage-specific mTOR KO mice with OA showed reduced apoptosis, induction of autophagy regulators, and decreased expression of the catabolic factor MMP13 (19). Increased receptor-interacting protein kinase-3 (RIP3), a marker of inflammatory cell death, accelerates inflammatory arthritis disease development *via* the TLR–TRIF–RIP3–IL-1β pivot, independently of MLKL (21). The activation (or expression) of RIP3 was markedly increased in damaged cartilage from OA. On the other hand, inhibition of RIP3 leads to a decrease in the arthritis incidence of OA (22). Therefore, targeting the mTOR-related autophagy and RIP3 kinase-mediated necroptosis may have therapeutic potential for OA.

Sodium butyrate is produced by the bacterial fermentation of dietary fibers (23, 24). Wang et al. showed that butyrate diminished the expression of catabolic markers such as MMP1, MMP3, and MMP13, which are induced by IL-1β. Notably, sodium butyrate ameliorated the disruption of type II collagen in OA chondrocytes (25, 26). Nevertheless, there has been little research on the effect of sodium butyrate on OA by the promotion of necroptosis as well as the underlying mechanisms.

*Lactobacillus acidophilus* LA-1 ameliorates OA by exerting antinociceptive and anti-inflammatory effects and preventing cartilage destruction (27). It has been reported that *L. acidophilus* produces SCFA, including butyrate, acetate, and propionate (28, 29), and butyrate has a therapeutic effect on the OA *via* regulating autophagy (18). We investigated the mechanism of *L. acidophilus* LA-1 and butyrate in an animal model of OA and in chondrocytes from OA patients. We also investigated the effect on the gut microbiota of orally administering *L. acidophilus* LA-1 and butyrate to mice with monosodium iodoacetate (MIA)-induced OA.

## Materials and methods

### Bacterial preparation

*Lactobacillus acidophilus* LA-1 (CNS Pharm Korea Co., Ltd. Seoul, Korea) was resuspended in phosphate-buffered saline (PBS) to a concentration of 125 mg/ml (2 × 10^11^ CFU/ml) (30).

### Animals

Seven-week-old male Wistar rats weighing 180–250 g at the start of the experiment were purchased from Central Lab Animal Inc. (Seoul, South Korea). Up to three animals per cage were housed in controlled rooms at 20–26°C and a 12-h light/dark cycle. Rats had *ad-libitum* access to a gamma-irradiated sterile diet (TD 2018S, Envigo, Indianapolis, IN, USA) and autoclaved reverse-osmosis water. All animal procedures were performed in accordance with the guidelines of the Laboratory Animal Welfare Act. The Catholic Medical School Institutional Animal Care and Use Committee (IACUC) Laboratory on animal care and use and rodent experimental guidelines and policies acquired Korea Excellent Animal Laboratory facility certification from the Catholic University of Korea IACUC and the Ministry of Food, Laboratory Animals and Songui Campus, and in 2017 and 2018, drug safety was officially certified (31).

### Monosodium iodoacetate-induced arthritis model and probiotics therapy

Before the start of the experiment, rats were randomly assigned to a treatment group and a control group. After being anesthetized with isoflurane, 7-week-old male Wistar rats (*n* = 6) were injected in their joints with 50 μl of MIA (Sigma-Aldrich, MO, USA) dissolved in saline. A 26.5-G needle was used for the administration, and injection was performed through the right knee ligament. Three days after the injection, the groups were separated and selected in consideration of the evaluation index of each individual based on pain and weight-bearing measurement values. MIA-induced OA mice were orally administered *L. acidophilus* (LA-1; CNS Pharm Korea Co., Ltd.) at 125 mg/ml (2 × 10^11^ CFU/ml) daily for 24 days. Sodium butyrate (Buty; Sigma-Aldrich) was orally administered at 200 mg/kg daily for 24 days. Celecoxib (positive control) was dissolved in 0.5% carboxymethylcellulose (CMC, Sigma-Aldrich) at 50 mg/kg and orally administered for 24 days (31).

### Assessment of pain behavior

Nociception in MIA-treated rats was tested using a dynamic plantar anesthesia meter (Ugo Basile, Gemonio, Italy). This device is an automated version of the von Frey hair evaluation procedure and is used to evaluate mechanical sensitivity. Assessments were performed by placing the rats on a metal mesh surface in an acrylic chamber, in a temperature-controlled room (20–26°C), and resting them for 10 min before placing a touch stimulator under each animal. Stimulus microfilaments (0.5 mm in diameter) were placed under the plantar surface of the hind paw using an adjustable angled mirror. When the device was activated, microscopic plastic monofilaments advanced at a constant rate and reached the foot in the proximal metatarsal region. The filaments applied a slowly increasing force to the plantar surface, starting below the sensing threshold and increasing until the stimulation was painful, as evidenced by debilitating rat paws. The force required to induce the paw withdrawal reflex was automatically recorded and measured in grams. Forces of up to 50 g and ramp rates of 25 s were used for all anesthesia tests (31).

### Assessment of weight bearing

Body weight balance in MIA-treated mice was analyzed using an incapacitance meter (IITC Life Sciences, CA, USA). Rats were acclimated for 5 min in an acrylic holder. After 5 min, both paws of the rat were fixed on the pad and the weight balance was measured for 5 s. Measurements were repeated three times in the same manner. The weights of the unguided and guided legs were determined and substituted into the formula to find the percentage value. The percentage value was calculated by comparing the leg with and without osteoarthritis (31).

### Differentiation of primary chondrocytes from the articular cartilage of OA patients

OA patients were recruited from the Orthopedic Surgery Department, Uijeongbu St. Mary’s Hospital, Seoul, South Korea (IRB No. UC14CNSI0150). Articular cartilage was acquired from patients undergoing replacement arthroplasty or joint replacement surgery. Cartilage was digested with 0.5 mg/ml of hyaluronidase, 5 mg/ml of protease type XIV, and 2 mg/ml of collagenase type V. Chondrocytes were incubated in Dulbecco’s modified Eagle’s medium (DMEM) with 10% fetal bovine serum (FBS) (31).

### Histopathological analysis

We obtained rat joint tissue and dorsal root ganglion (DRG) 4 weeks after MIA induction. For rat joint tissue and colon tissue, 5-μm paraffin-embedded tissue sections were subjected to paraffin removal, hydration, and dehydration, followed by Safranin O staining. Dorsal root ganglion tissue was cryo-sectioned at 5 μm, mounted on slides, and subjected to pathological analysis.

### Immunohistochemistry

For dorsal root ganglion immunohistochemistry (IHC), anti-transient receptor potential cation channel subfamily V member 1 (TRPV1; 1:200, Cat. No. NB100-1617, Novus, Centennial, CO, USA) and anti-calcitonin gene-related peptide (CGRP; 1:400, Cat. No. ab81887, Abcam, Cambridge, UK) were incubated at 4°C overnight. For articular joint IHC, anti-interleukin-1β (IL-1β; 1:400, Cat. No. NB600-633, Novus, Centennial, CO, USA), anti-matrix metalloproteinase 13 (MMP13; 1:200, Cat. No. ab39012, Abcam, Cambridge, UK), anti-tumor necrosis factor α (TNF-α; 1:150, Cat. No. ab6671, Abcam, Cambridge, UK), anti-monocyte chemoattractant protein 1 (MCP-1; 1:600, Cat. No. ab7202, Abcam, Cambridge, UK), inducible nitric oxide synthase (iNOS; 1:100, Cat. No. ab15323, Abcam, Cambridge, UK), IL-17 (1:400, Cat. No. ab79056, Abcam, Cambridge, UK), phosphorylated-signal transducer and activator of transcription 3 serine 727 (p-STAT3 ser727; 1:100, Cat. No. ab30647, Abcam, Cambridge, UK), Forkhead box P3 (Foxp3; 1:400, Cat. No. NB100-39002, Novus, Centennial, CO, USA), receptor-interacting serine/threonine-protein kinase 1 (RIPK1; 1:400, Cat. No. #PA5-20811, Invitrogen), receptor-interacting serine/threonine-protein kinase 3 (RIPK3; 1:200, Cat. No. #PA5-19956, Invitrogen), phosphorylated-mixed linage kinase domain-like protein S345 (p-MLKL S345; 1:250, Cat. No. ab196436, Abcam, Cambridge, UK), and G-protein-coupled receptor 43 (GPR43; 1:100, Cat. No. MBS541594, MyBioSource, San Diego, CA, USAMyBioSource, San Diego, CA, USA) were incubated at 4°C overnight. For large-intestine IHC, anti-tumor necrosis factor α (TNF-α; 1:150, Cat. No. ab6671, Abcam, Cambridge, UK), anti-MCP-1 (1:400, Cat. No. ab7202, Abcam, Cambridge, UK), and the same anti-IL-1β (IL-1β; 1:400, Cat. No. NB600-633, Novus, Centennial, CO, USA) that IHC was tested in the joints were used and incubated overnight at 4°C.

### Immunofluorescence

For large-intestine immunofluorescence (IF), unconjugated large intestine-1 (ZO-1; 1:400, Cat. No. 40-2200, Invitrogen) and Alexa Fluor 488-conjugated occludin (OCLN; 1:400, Cat. No. #331588, Invitrogen) were used for the primary culture, and ZO-1 was incubated using a goat anti-rabbit PE secondary antibody (Cat. No. 4050-09, 1:200, Southern Biotech, Birminham, AL, USA). Nuclear staining was performed using DAPI, and fluorescence analysis was performed using ZEN2012 (Blue Edition; Zeiss, Oberkochen, Germany) software. For the IF of chondrocyte autophagy, unconjugated sequestosome-1 (p62; 1:200, Cat. No. ab56416, Abcam, Cambridge, UK), unconjugated autophagy marker light chain 3B (LC3B; 1:400, Cat. No. ab48394, Abcam, Cambridge, UK), and PE-conjugated-bound membrane protein 1 (LAMP-1; Cat. No. sc-20011 PE, Santa Cruz, Dallas, TX, USA) were cultured. Primary antibody incubation was performed overnight at 4°C, and secondary antibody incubation involved p62-binding anti-mouse FITC and LC3B-binding anti-rabbit APC. Secondary antibody incubation in an unconjugated antibody was performed at room temperature for 2 h.

### Fecal DNA extraction, PCR amplification, and sequencing

Individual rat fecal samples on ice were sent immediately to the research site after collection in a plastic container and stored at −70°C within 12 h of arrival. Total DNA was extracted using the FastDNA^®^ SPIN Kit for Soil (MP Biomedicals, USA), in accordance with the manufacturer’s instructions. PCR amplification was performed using fusion primers targeting from V3 to V4 regions of the 16S rRNA gene with the extracted DNA. For bacterial amplification, fusion primers of 341F (5′-AATGATACGGCGACCACCGAGATCTACAC-XXXXXXXX-TCGTCGGCAGCGTC-AGATGTGTATAAGAGACAG-CCTACGGGNGGCWGCAG-3′; the underlined sequence indicates the target region primer) and 805R (5′-CAAGCAGAAGACGGCATACGAGAT-XXXXXXXX-GTCTCGTGGGCTCGG-AGATGTGTATAAGAGACAG-GACTACHVGGGTATCTAATCC-3′). The fusion primers were constructed in the following order: P5 (P7) graft binding, i5 (i7) index, Nextera consensus, sequencing adaptor, and target region sequence. The amplifications were carried out under the following conditions: initial denaturation at 95°C for 3 min, followed by 25 cycles of denaturation at 95°C for 30 s, primer annealing at 55°C for 30 s, and extension at 72°C for 30 s, with a final elongation at 72°C for 5 min. The PCR product was confirmed by using 1% agarose gel electrophoresis and visualized under a Gel Doc system (Bio-Rad, Hercules, CA, USA). The amplified products were purified with the CleanPCR (CleanNA, Waddinxveen, Netherland). Equal concentrations of purified products were pooled together and removed short fragments (non-target products) with CleanPCR (CleanNA, Waddinxveen, Netherland). The quality and product size were assessed on a Bioanalyzer 2100 (Agilent, Palo Alto, CA, USA) using a DNA 7500 chip. Mixed amplicons were pooled and the sequencing was carried out at Chunlab, Inc. (Seoul, South Korea), with the Illumina MiSeq Sequencing system (Illumina, USA) according to the manufacturer’s instructions.

### Data analysis pipeline

The processing of raw reads started with quality checking and filtering of low quality (32). After the QC pass, paired-end sequence data were merged together using fastq_mergepairs command of VSEARCH version 2.13.4 (33) with default parameters. Primers were then trimmed with the alignment algorithm of Myers and Miller (34) at a similarity cutoff of 0.8. Non-specific amplicons that do not encode 16S rRNA were detected by nhmmer (35) in HMMER software package ver. 3.2.1 with hmm profiles. Unique reads were extracted and redundant reads were clustered with the unique reads by the derep_fulllength command of VSEARCH (33). The EzBioCloud 16S rRNA database (36) was used for taxonomic assignment using the usearch_global command of VSEARCH (33) followed by a more precise pairwise alignment (34). Chimeric reads were filtered on reads with <97% similarity by reference-based chimeric detection using the UCHIME algorithm (37) and the non-chimeric 16S rRNA database from EzBioCloud. After chimeric filtering, reads that were not identified to the species level (with <97% similarity) in the EzBioCloud database were compiled, and cluster_fast command2 was used to perform *de-novo* clustering to generate additional OTUs. Finally, OTUs with single reads (singletons) were omitted from further analysis. All analytics mentioned above were performed in EzBioCloud 16S-based MTP, which is ChunLab’s bioinformatics cloud platform. Also, raw 16S rRNA sequences were bioinformatically analyzed using QIIME 2 version 2019.4 (38), as previously described (39).

### Real-time polymerase chain reaction and quantitative polymerase chain reaction

Whole ribonucleic acid (RNA) was separated from chondrocytes using the TRI reagent protocol (Invitrogen). Complimentary DNA (cDNA) was generated by reverse transcription of single-stranded ribonucleic acid using the High-Capacity cDNA Reverse Transcription Kit (Applied Biosystems, Waltham, MA, USA) as per the manufacturer’s instructions. mRNA was subjected to real-time PCR using the Light-Cycler FastStart DNA Master SYBR Green I Kit (TaKaRa, Kusatsu, Japan) in accordance with the manufacturer’s directions. Expression levels were normalized relative to β-actin. The sequences of the primers used are listed in the **Supplementary Table**. PCR amplification was performed using the Light-Cycler Real-Time PCR System (Roche Diagnostics, Indianapolis, IN, USA).

### Western blotting

Chondrocytes (1 × 10^6^ cells/well) were treated with IL-1β 20 ng/ml (R&D Systems, MN, USA) and 1 mM of sodium butyrate (Buty; Sigma-Aldrich) for 24 h. Proteins isolated from cells were resolved by SDS-PAGE and transferred to nitrocellulose membranes (Amersham Pharmacia Biotech, Piscataway, NJ, USA), followed by Western blotting using SNAP IDs. An enhanced chemiluminescence (ECL) detection kit (Thermo Fisher Scientific) was used with the following antibodies: anti-phosphorylated MLKL (1:1,000), anti-MLKL (1:1,000), and anti-GAPDH (1:2,000). The primary antibodies to MLKL, p-MLKL, and GAPDH were diluted in 0.1% skim milk in Tris-buffered saline Tween-20 and incubated for 15 min at room temperature. The membrane was washed and incubated with horseradish peroxidase-conjugated secondary antibody for 10 min at room temperature. Western blot bands were quantitatively analyzed using Fiji/ImageJ software.

### Flow cytometry

Intracellular staining was performed as described previously (40). Splenocytes and peripheral blood mononuclear cells (PBMCs) were stained with the following antibodies: PE-Cy 7-CD4 (#201516; BioLegend, San Diego, CA, USA), FITC-CD25 (#202103; BioLegend), PE-Foxp3 (#12-5773-82; eBioscience, San Diego, CA, USA), FITC-IFN-γ (#507804; BioLegend), PE-Cy5.5-IL-17A (#45-7177-82, eBioscience), and PE-IL-10 (#555088, BD Biosciences, Franklin Lakes, NJ, USA). Events were analyzed using FlowJo software (Tree Star, Ashland, OR, USA) and CytExpert experiment-based software (Beckman Coulter, Inc, Bera, CA, USA.).

### Statistical analysis

Statistical analysis was performed using Prism (version 8.01; GraphPad Software, San Diego, CA, USA). The significance of differences among groups was calculated by generalized one-way ANOVA. For the pain and weight-bearing data, a generalized two-way ANOVA was performed, and a between-group analysis by period was conducted. The significance of differences between groups was assessed by Bonferroni *post-hoc* test if significant differences were observed between groups. Numerical data were compared between two groups by the Mann–Whitney test and an unpaired *t*-test. Data are means ± standard error of the mean. A *p*-value of 0.05 was considered indicative of statistical significance.

## Results

### Intestinal function is impaired, and the gut microbiome is altered in rats with MIA-induced OA

**Figure 1:**
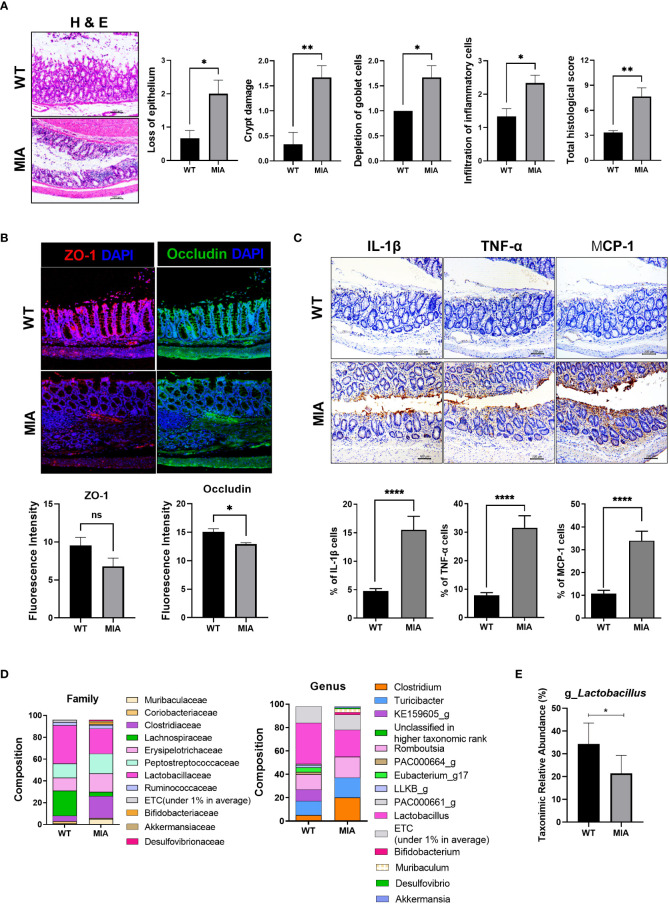
Osteoarthritis (OA) leads to an imbalanced intestinal environment and an altered microbiome. **(A)** Intestinal epithelial tissue images of the healthy (WT) and diseased (monosodium iodoacetate, MIA) groups at 3 weeks after OA induction (magnification ×200). **(B)** Intestinal tight junction protein expression (magnification ×200). **(C)** Expression of inflammatory factors by immunohistochemistry (IHC) (magnification ×200). **(D)** The microbiome of fecal samples in WT and MIA. **(E)** The abundance of *Lactobacillus* in WT and MIA. Data are means ± SD (*p < 0.05, **p < 0.01, ****p < 0.001), ns, not significant.

Histochemical staining showed severe damage to the intestine in the OA group compared to the wild-type group (**Figure 1A**). The expression of ZO-1 and occludin tended to decrease in OA subjects (**Figure 1B**). IHC showed that the intestinal expression of IL-1β, TNF-α, and MCP-1 (aka CCL2) was significantly increased in the OA group (**Figure 1C**). In terms of the fecal microbiome, *Lactobaillaceae* (family level) and *Lactobacillus* (genus level) were decreased in the OA group (**Figure 1D**). Furthermore, *Peptostreptococcaceae*, frequently present in colorectal cancer patients, showed a tendency to increase. *Eubacterium*, a producer of SCFA, was detected in the wild-type group, but not in the OA group. In addition, *Desulfovibrio*, which negatively affects the intestinal environment, slightly increased in the OA group. At the genus level, lactic acid bacteria decreased in the OA group (**Figure 1E**).

### LA-1 reduces the pain and joint destruction associated with OA

**Figure 2:**
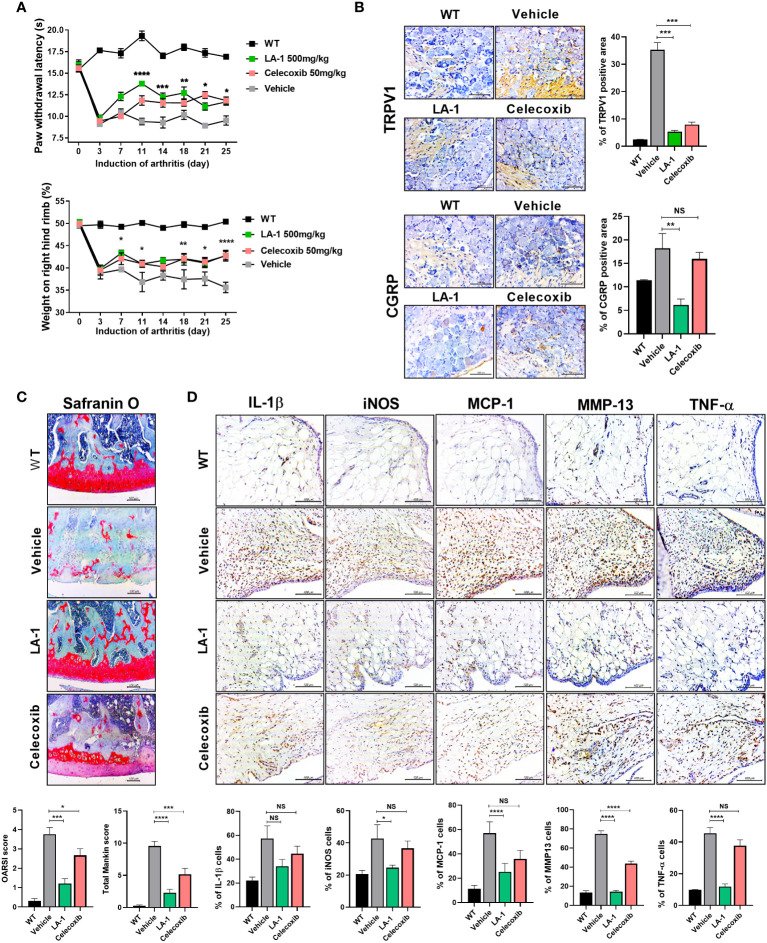
Supplementation of live *Lactobacillus acidophilus* (LA-1) reduces OA-mediated pain and inflammation. **(A)** Pain withdrawal latency and weight on the right hind limb were measured for 25 days in the WT, vehicle, LA-1, and celecoxib groups. **(B)** IHC of TRPV1 and CGRP in the dorsal root ganglion (DRG) of rats treated with LA-1 for 3 weeks. **(C)** After 3 weeks of MIA injection, OA-induced knee joints were sectioned and subjected to Safranin O staining. The OARSI score and total Mankin score were calculated (magnification ×200). **(D)** Analysis of positive cell expression in the synovial membrane of the same tissue for inflammatory factors (IL-1β, iNOS, MCP-1, TNF-α) and catabolic factors (MMP13), accompanying the occurrence of arthritis, was performed by IHC (magnification ×400). Data are means ± SD (*p < 0.05, **p < 0.01, ***p < 0.005, ****p < 0.001), ns, not significant.

Inactivated LA-1 ameliorates OA (30). To investigate the effect of live LA-1 on OA, we injected live LA-1 once daily for 3 weeks into rats with MIA-induced OA. The PWL and weight bearing increased significantly in the inflamed hind paw of rats given live LA-1 (50 mg/kg) compared with vehicle-treated OA rats (**Figure 2A**). DRG was isolated from the rat spine and IHC was conducted using the neurotransmitter markers, transient receptor potential cation channel subfamily V member 1 (TRPV1) and calcitonin gene-related peptide (CGRP) (**Figure 2B**). CGRP and TRPV1 expression was decreased in the DRG, resulting in a reduction of pain. Safranin O staining of the joints was assessed using the tissue score index, which comprises the OARSI score and total Mankin score. Cartilage destruction was markedly decreased in the LA-1-treated group compared to the vehicle group (**Figure 2C**). By IHC, the levels of the inflammatory markers, IL-1β, iNOS, MCP-1, MMP13, and TNF-α, were decreased in the LA-1 group (**Figure 2D**). IHC results showed that the expression of Foxp3 was increased and that of IL-17 and its transcription factor STAT3 phosphorylation was decreased by LA-1 (**Supplementary Figure 1A**). Also, Th1 (CD4^+^IFN-γ^+^) and Th17 (CD4^+^IL-17^+^) cells were decreased, and IL-10^+^ and Treg (CD4^+^CD25^+^FOXP3^+^) cells were increased in splenocytes and PBMC by LA-1 (**Supplementary Figures 1B, 2**). Therefore, live LA-1 can improve joint destruction and inflammation *in vivo*.

### LA-1 ameliorates OA-associated intestinal epithelial tissue damage and microbiome imbalance

**Figure 3:**
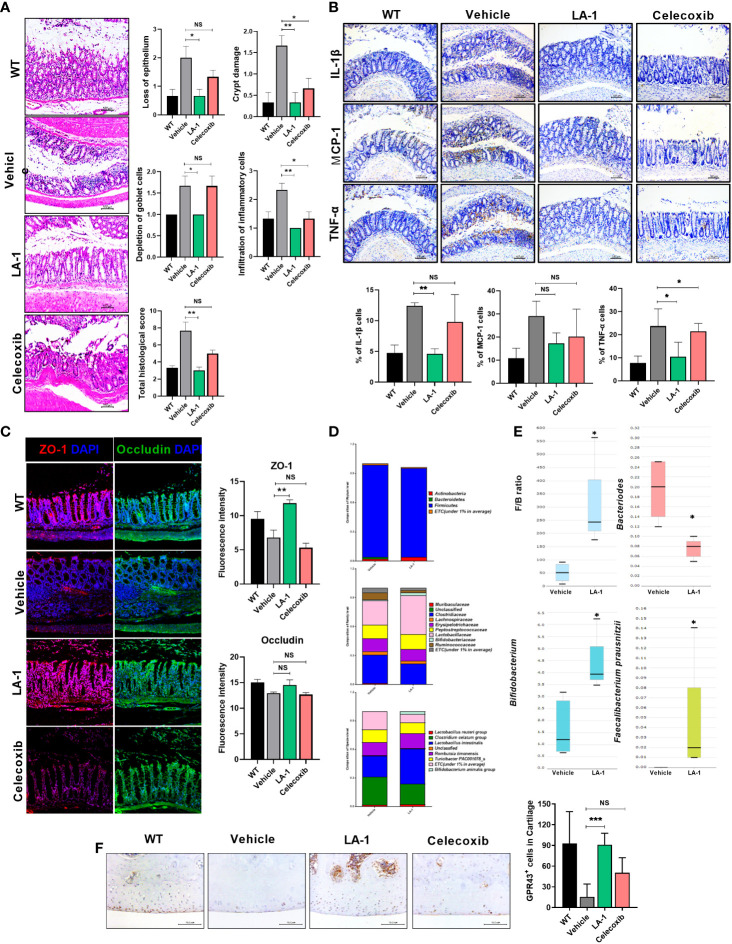
LA-1 improves the imbalanced intestinal environment and microbiome. **(A)** Representative images show H&E staining of the intestine in the WT, vehicle, LA-1, and celecoxib groups (magnification ×200). **(B)** Inflammatory factor expression in intestinal epithelial cells was determined by IHC in each group (magnification ×200). **(C)** Tight junction protein (TJP) expression in intestinal epithelial cells was increased by LA-1 (magnification ×200). **(D)** The feces of OA rats had different phyla, family, and species phenotypes from those treated with LA-1, and a relatively high proportion of beneficial bacteria was found in the feces of LA-1-treated rats. **(E)** LA-1 altered the F/B ratio and the population of butyrate-producing bacteria. **(F)** LA-1 increased the level of the short-chain fatty acid receptor G-protein-coupled receptor 43 (magnification ×400). Data are means ± SD (*p < 0.05, **p < 0.01, ***p < 0.005), ns, not significant.

There is a relationship between OA and the gut environment (10, 15, 16, 30). We found intestinal inflammation during OA progression (**Figure 1A**). Therefore, we hypothesized that inflammatory markers exacerbate OA and migrate to the inflamed intestine. Pathological analysis of the intestine showed that intestinal damage was reduced in the LA-1 group (**Figure 3A**). In addition, IHC showed that the levels of IL-1β, MCP-1, and TNF-α (associated with intestinal inflammation) decreased (**Figure 3B**) by LA-1. In addition, the TJP markers OCLN and ZO-1 were increased to above the normal range by LA-1 (**Figure 3C**). The abundance of *Bifidobacterium* and *Faecalibacterium prausnitzii*, butyrate producers, increased, and the *Firmicutes*-to-*Bacteroidetes* ratio was altered (**Figures 3D, E**). Moreover, when SCFA is directly or indirectly increased by LA-1, the expression of the receptors, including G-protein-coupled receptor 43 (GPCR43 or GPR43 also known as FFAR2) and GPR109a, accepting SCFA, is also important (26). In the joint cavity, GPR43 and GPR109a expression was increased in the LA-1 group compared to the vehicle group (**Figure 3F**, **Supplementary Figure 3**). Also, we found that the expression of anabolic factors in OA patient-derived primary chondrocytes, such as TIMP1, TIMP3, ACAC, and COL2A1, was increased by LA-1 (**Supplementary Figure 4**). We hypothesized that butyrate from butyrate producers (*F. prausnitzii*, *Bifidobacterium*) regulated by LA-1 supplementation would control the progression of OA.

### Butyrate reduces pain caused by osteoarthritis and has chondroprotective effects and intestinal tight junction maintenance and increased effect

**Figure 4:**
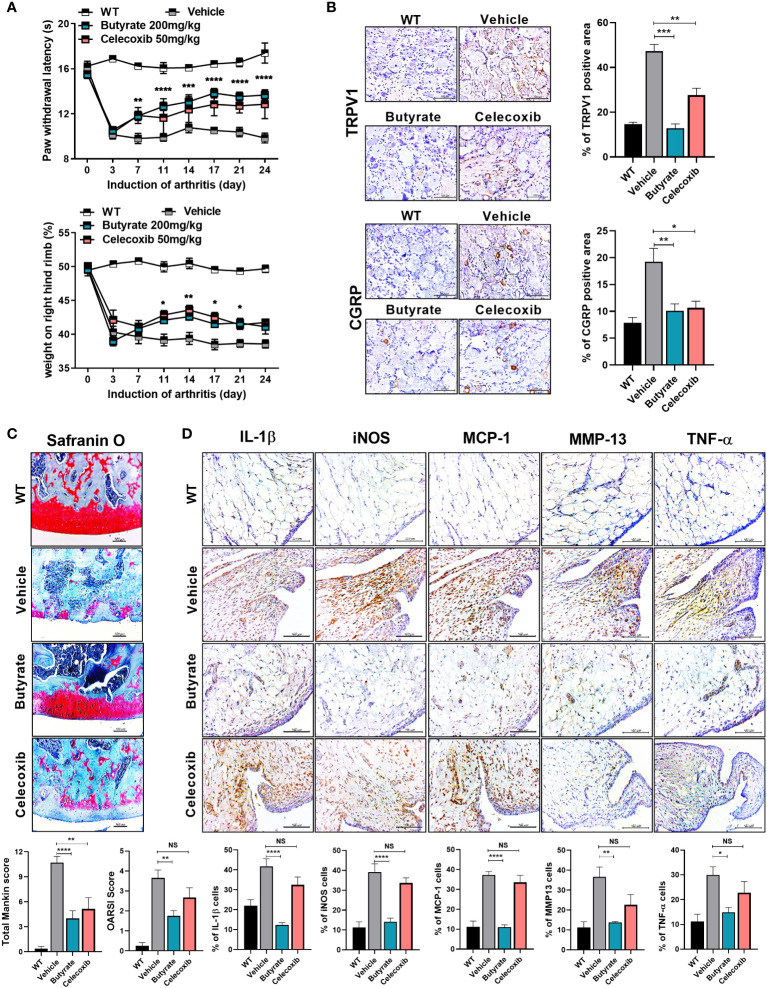
Butyrate reduces OA pain and exerts a chondroprotective effect. **(A)** Butyrate improved rat paw withdrawal latency and weight bearing at 3 weeks after induction of MIA, indicating pain suppression. **(B)** Butyrate reduced the levels of neurotransmitters in rat dorsal root ganglion (magnification ×400). **(C)** Three weeks after OA induction, butyrate protected the rat knee joint from OA-associated degradation (magnification ×200). **(D)** IHC analysis confirmed the regulation of butyrate-mediated inflammatory factors (IL-1β, iNOS, MCP-1, TNF-α) and catabolic factors (MMP13) in the same synovial tissue site as determined by IHC (magnification ×400). Data are means ± SD (*p < 0.05, **p < 0.01, ***p < 0.005, ****p < 0.001), ns, not significant.

**Figure 5:**
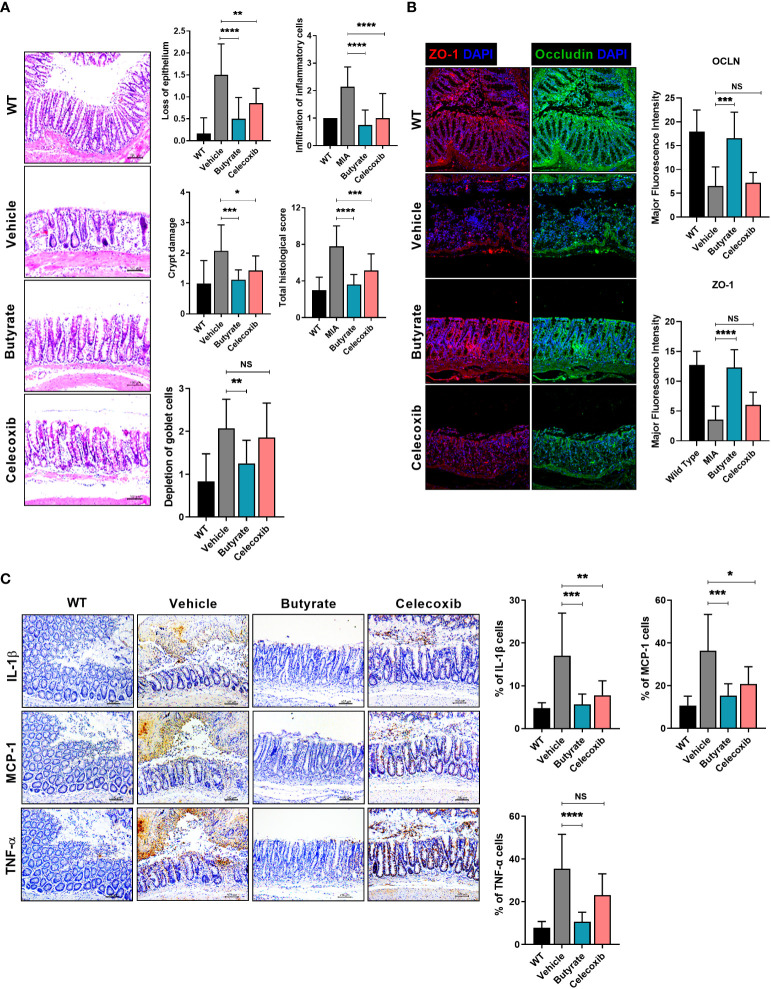
Butyrate restores tight junction proteins, inhibits intestinal damage, and reduces inflammatory factor levels. **(A)** Three weeks after MIA injection, the degree of intestinal damage in rats was evaluated by H&E staining (magnification ×200). **(B)** Effect of butyrate on tight junction protein (TJP) expression by immunofluorescence staining (magnification ×200). **(C)** Effect of butyrate on the expression levels of inflammatory factors in rat intestine by IHC (magnification ×200). Data are means ± SD (*p < 0.05, **p < 0.01, ***p < 0.005, ****p < 0.001), ns, not significant.

The administration of butyrate to OA animals improved the PWL and body weight ratio in both feet (**Figure 4A**). In addition, TRPV1 and CGRP, pain signaling-related genes, were decreased in the DRG, where the knee joint pain signal is transduced (**Figure 4B**). The OARSI score and total Mankin score were the lowest in the butyrate group (**Figure 4C**). Also, butyrate decreased the levels of inflammatory factors, including IL-1β, MCP-1, MMP13, and TNF-α (**Figure 4D**). In particular, iNOS, which is associated with oxidative stress and regulated by SCFAs, was also decreased by butyrate (**Figure 4D**). IHC showed that the expression of Foxp3 was increased and that of IL-17 and its transcription factor STAT3 phosphorylation was decreased by butyrate (**Supplementary Figure 5A**). Also, Th1 (CD4^+^IFN-γ^+^) and Th17 (CD4^+^IL-17^+^) cells were decreased, and IL-10^+^ and Treg (CD4^+^CD25^+^FOXP3^+^) cells were increased in splenocytes and PBMC by butyrate (**Supplementary Figure 5B**). Next, we investigated the effect of butyrate on the intestinal environment. Surprisingly, like LA-1, butyrate improved the morphology of the intestinal tissue. Intestinal histological scores were decreased by butyrate (**Figure 5A**). In addition, the expression of TJP markers, such as OCLN and ZO-1, was increased by butyrate (**Figure 5B**). Furthermore, IL-1β, MCP-1, and TNF-α expression levels were markedly reduced by butyrate (**Figure 5C**). Therefore, butyrate reduced the levels of inflammatory factors and increased the binding of intestinal epithelial cells, thereby enhancing the intestinal environment.

### Butyrate modulates the levels of inflammatory mediators, anabolic factors, and catabolic factors in OA chondrocytes

**Figure 6:**
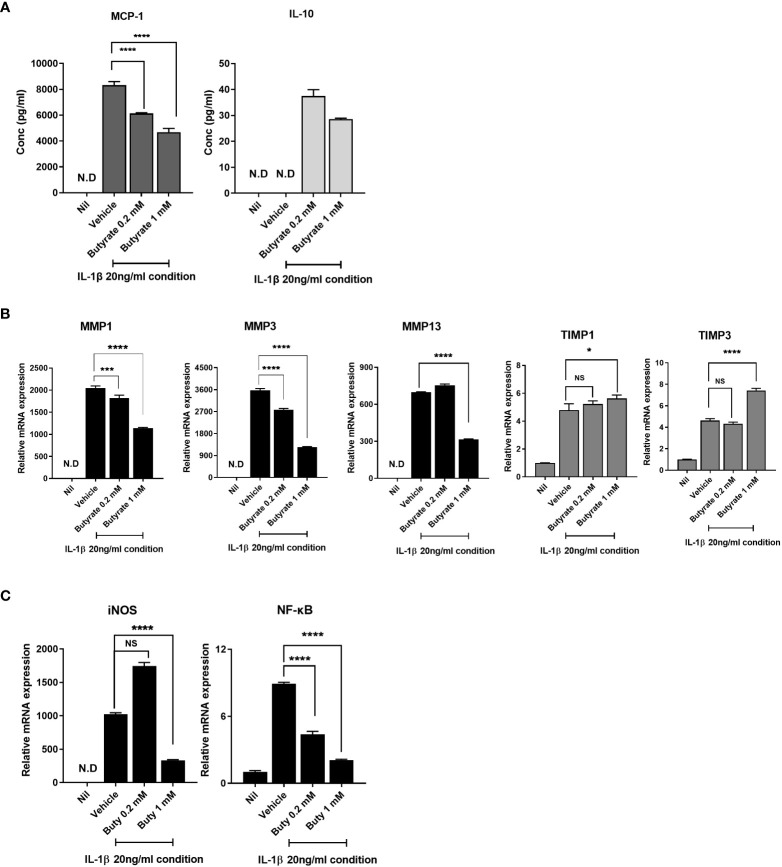
Butyrate rebalances anabolic/catabolism and suppresses the expression of inflammatory factors to prevent exacerbation of OA. **(A)** Protein levels of monocyte chemoattractant protein 1 (MCP-1) and interleukin 10 (IL-10) in chondrocytes cultured in serum-free medium with 20 ng/ml of IL-1β for 24 h. **(B)** To determine the level of anabolic/catabolism expressed from chondrocytes upon butyrate treatment within an inflammatory environment, quantitative PCR was performed to determine mRNA levels. **(C)** Inducible nitric oxide synthase (iNOS) and nuclear factor kappa-light-chain-enhancer of activated B-cell (NF-κB) expression levels of chondrocytes under inflammatory conditions were analyzed by quantitative PCR. Data are means ± SD (*p < 0.05, ***p < 0.005, ****p < 0.001), nd, not detected; ns, not significant.

Next, we evaluated the effect of butyrate on the expression of anabolic, catabolic, and anti-inflammatory markers in human OA chondrocytes. Butyrate significantly downregulated inflammatory cytokines, such as MCP-1, in IL-1β-stimulated chondrocytes from OA patients (20 ng/ml). However, the expression of the anti-inflammatory cytokine, IL-10, was increased by butyrate (**Figure 6A**). Butyrate decreased the MMP1, MMP3, and MMP13 mRNA levels in OA chondrocytes but significantly increased those of TIMP1 and TIMP3 (**Figure 6B**). Also, butyrate decreased iNOS and NF-κB expression (**Figure 6C**). Also, TSA, which is known as an HDAC inhibitor, decreased the expression of NF-κB (**Supplementary Figure 6**). These results suggest that butyrate exerts a protective effect on cartilage.

### Butyrate inhibits OA progression by preventing chondrocyte inflammatory cell death and restoring autophagy

**Figure 7:**
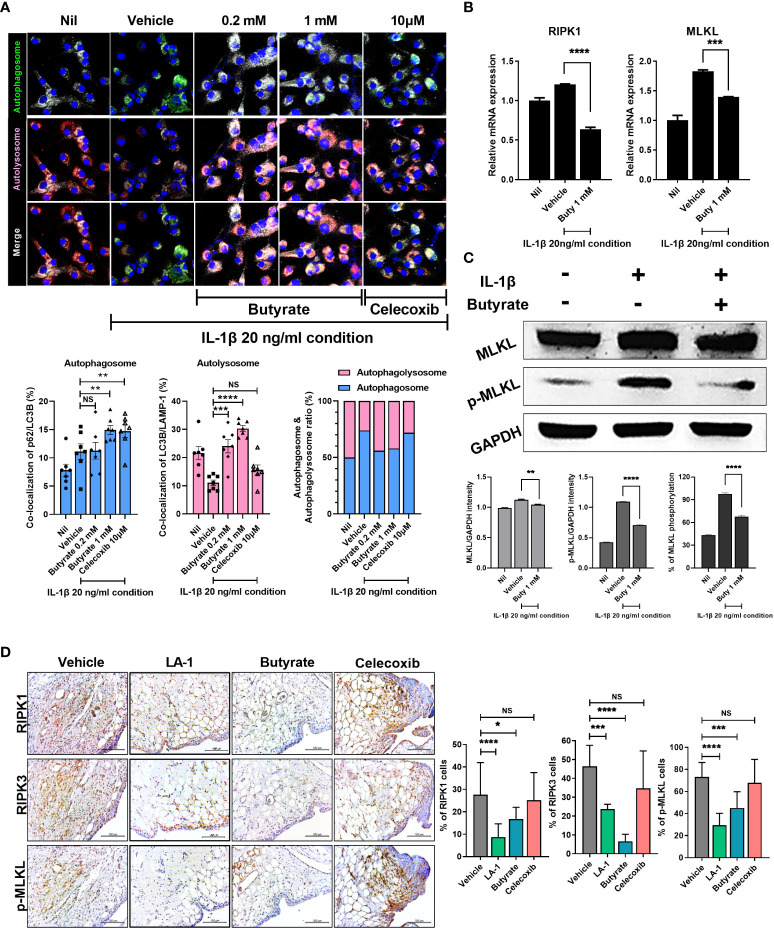
Butyrate reduced inflammatory cell death by restoring abnormal autophagy of chondrocytes in an inflammatory environment. **(A)** Effect on autophagy of butyrate in chondrocytes cultured in medium with IL-1β at 20 ng/ml (magnification ×200). **(B)** Effect of butyrate on the expression levels of necrosis factors of chondrocytes under inflammatory conditions. **(C)** In chondrocytes, the effect of butyrate on MLKL phosphorylation in chondrocytes under inflammatory conditions. **(D)** IHC showed that live LA-1 and butyrate modulated the levels of necroptosis factors in OA synovial tissue (magnification ×400). Data are means ± SD (*p < 0.05, **p < 0.01, ***p < 0.005, ****p < 0.001), ns, not significant.

Dysregulation of autophagy reportedly induces OA (41). To investigate the role of butyrate in autophagy, we evaluated the autophagosome based on co-localization of p62 (SQSTM1) and LC3b and the autolysosome based on co-localization of LC3b and LAMP-1. In the Nil condition, the number of autophagosomes increased slightly due to prestarvation, but the autolysosome numbers were unchanged. Under stimulation conditions, stimulation-induced autophagy induction was increased in the vehicle, whereas autolysosome levels were decreased, which means an impaired autophagic process. However, butyrate administration led to improve the autophagic process by increasing the ratio of autolysosome (**Figure 7A**). In chondrocytes, the mRNA levels of RIPK1 and MLKL, necroptosis factors, increased under inflammatory conditions; butyrate reduced the mRNA levels of these necroptosis factors (**Figure 7B**). In addition, the protein expression level of phosphorylated MLKL was significantly reduced by butyrate (**Figure 7C**). Also, TSA decreased phosphorylated MLKL, RIPK1, and RIPK3 (**Supplementary Figure 7**). The *in-vivo* histopathology findings were confirmed by IHC. IHC showed that both live LA-1 and butyrate decreased the levels of necroptosis factors in joint synovial tissue (**Figure 7D**). Our results indicated that butyrate can be a therapeutic utility in OA by decreasing inflammatory cell death followed by improving the autophagic process.

## Discussion

Body weight, gender, and heredity are risk factors for OA, the most frequent arthritis of middle age. There is no cure for OA, which is typically treated with analgesics and anti-inflammatory agents such as corticosteroids and non-steroidal anti-inflammatory drugs (NSAIDs) (42, 43). Although these medications decrease pain, they have several safety concerns. Steroids can induce a slew of side effects, making long-term use problematic, and NSAIDs can cause gastrointestinal issues and hepatotoxicity.

Microorganisms are abundant in the gastrointestinal system (gut microbiota) and oral cavity (oral microbiota) (44). Several studies on the therapeutic effect of probiotics in RA, an inflammatory and autoimmune illness, have been conducted. In contrast, few works have focused on the therapeutic effect of probiotics in OA. Because OA involves persistent low-grade inflammation and the therapeutic effect of probiotics can be predicted using the gut–joint axis notion, more research into this topic is required.

Several probiotic strains have been examined in OA. Studies of *Lactobacillus casei* Shirota demonstrated its effects and mechanism of action in experimental OA and placebo-controlled clinical trials. The oral treatment of *L. casei* Shirota reduces the serum hs-CRP level of OA patients (45). We reported that inactivated *L. acidophilus* was effective for MIA-induced OA. In this study, inactivated *L. acidophilus* reduced OA-related pain and postponed disease progression by inhibiting proinflammatory cytokine production and reducing cartilage degradation (27). The oral administration of *Streptococcus thermophilus* reduces OA-related degeneration in the knee (46).

It is well known that probiotic bacteria produce essential vitamins and SCFA, including acetate, propionate, and butyrate, which have an impact on the health of the host. Zhang et al. reported the correlation between probiotics, including *Lactobacillus*, and SCFA, including butyrate (47). Also, it has been reported that *Lactobacillus* produces butyrate and increases butyrate-producing bacteria, and increasing butyrate has a therapeutic effect (48). In OA mice, the abundance of *Lactobacillus* in the gut was decreased. Tissue destruction was improved, and the expression levels of inflammatory cytokines were decreased by live LA-1 compared to the vehicle. Intestinal destruction was markedly improved by live LA-1. Also, live LA-1 induced to increase the level of *Faecalibacterium* which produces butyrate. Our findings are consistent with prior studies on the gut–joint axis. Next, we investigated the effect of butyrate on the development and progression of OA. Butyrate decreased the OARSI score and total Mankin score of the knee joint compared to vehicle. Butyrate also significantly decreased the expression levels of inflammatory cytokines and improved intestinal destruction. We also found that administration of LA-1 and butyrate increased the expression of GPR43 and GPR109a which are receptors accepting SCFA. Luo et al. and Wu et al. reported increasing GPR43 (49) and GPR109a (50) expression by butyrate administration. These data imply that the therapeutic effect of LA-1 seems to be due to the increased LA-1-producing butyrate or the increased butyrate-producing bacteria by LA-1, although we did not measure the SCFA level.

Autophagy is a cellular and molecular self-degradation system that eliminates unwanted or defective proteins and organelles (51). Autophagy dysfunction is linked to disease development and progression (52, 53). Autophagy is reportedly impaired in OA animal models and OA patients (18, 31, 54). Autophagy requires the formation of autophagosomes and autolysosomes at particular ratios (31). We reported that the accumulation of autophagosomes leads to an increase in the risk of necroptosis and acts as a pathogen on the OA. It has been reported that SCFA induced autophagy (55). The AMPK and mTOR signaling pathways are well known to induce autophagy (56). Butyrate treatment increased autolysosome formation and expression of AMPK, and inhibition of AMPK decreased butyrate-induced autophagy (57). Zhou et al. reported that butyrate ameliorates OA progression and development by recovering impaired autophagy *via* targets of the mTOR pathway, including PI3K/Akt (18). Also, many studies revealed that autophagy decreases inflammatory cell death (necrosis). Lim et al. reported that autophagy decreases necrosis and impaired autophagy enhances TNF-mediated necrosis (58). In this study, we revealed that butyrate promoted autophagy and decreased inflammatory cell death.

In this study, we used a chemical (MIA)-induced OA animal model. The MIA model may not be a perfect model for understanding the mechanisms of human OA development and progression. This limitation, however, does not significantly alter the major conclusions of this study, which showed that LA-1 and butyrate had beneficial effects on pain, cartilage histology, and molecular markers in our OA model.

In our experimental OA animal model, LA-1 and butyrate significantly decreased joint pain by desensitizing nociception, lowering the levels of pain modulators and proinflammatory factors. LA-1 and butyrate effectively maintained joint tissue integrity as the OA illness progressed, and the gut flora of OA animals underwent considerable changes as a result. Finally, the administration of LA-1 and butyrate ameliorates OA symptoms by improving autophagy and inhibiting inflammatory cell death. Our data showed the therapeutic potential of LA-1 and butyrate for OA treatment.

## Data availability statement

The datasets presented in this study can be found in online repositories. The names of the repository/repositories and accession number(s) can be found below: https://www.ncbi.nlm.nih.gov/, PRJNA806482.

## Ethics statement

The studies involving human participants were reviewed and approved by The Institutional Inspection Board of Uijeongbu St. Mary’s Hospital (UC14CNSI0150). The patients/participants provided their written informed consent to participate in this study. The animal study was reviewed and approved by The Animal Research Ethics Committee of the Catholic University of Korea (2020-0037-02, 2020-0264-01).

## Author contributions

Conception and design: M-LC. Analysis and interpretation of data: K-HC, HSN, JJ, JSW, ARL, SYL, JSL, and IGU. Drafting of the article: K-HC, JJ, and JSW. Editing of the manuscript: SJK, SHP, and M-LC. M-LC takes responsibility for the integrity of the work in its entirety. All authors contributed to the article and approved the submitted version.

## Funding

This research was supported by a grant from the Korea Health Technology R&D Project through the Korea Health Industry Development Institute (KHIDI), funded by the Ministry of Health & Welfare, Republic of Korea (grant number HI20C1496).

## Conflict of interest

The authors declare that the research was conducted in the absence of any commercial or financial relationships that could be construed as a potential conflict of interest.

## Publisher’s note

All claims expressed in this article are solely those of the authors and do not necessarily represent those of their affiliated organizations, or those of the publisher, the editors and the reviewers. Any product that may be evaluated in this article, or claim that may be made by its manufacturer, is not guaranteed or endorsed by the publisher.
