# Supplementary material for: Lactobacillus (LA-1) and butyrate inhibit osteoarthritis by controlling autophagy and inflammatory cell death of chondrocytes
Source: Front Immunol. 2022 Oct 17;13:930511. doi: 10.3389/fimmu.2022.930511 (PMC9619036; doi:10.3389/fimmu.2022.930511)
Supplement: Supplementary file 1 [file DataSheet_1.pdf]

***Lactobacillus* (LA-1) and Butyrate inhibit Osteoarthritis by controlling Autophagy and Inflammatory Cell Death of Chondrocytes**

Keun-Hyung Cho[1, 2], Hyun Sik Na[1, 2], JooYeon Jhun[1], Jin Seok Woo[1], A Ram Lee[1, 2], Seung Yoon Lee[1,2], Jeong Su Lee[1, 2], In Gyu Um[1,2], Seok Jung Kim[3], Sung-Hwan Park[4], Mi-La Cho[1, 5]

[1]Rheumatism Research Center, Catholic Research Institute of Medical Science, Catholic University of Korea, Seoul 06591, Korea

[2]Department of Biomedicine & Health Sciences, College of Medicine, Catholic University of Korea, Seoul 06591, Korea

[3]Department of Orthopedic Surgery, College of Medicine, Catholic University of Korea, Seoul 06591, Korea

[4]Division of Rheumatology, Department of Internal Medicine, Seoul St. Mary's Hospital, College of Medicine, Catholic University of Korea, Seoul 06591, Korea

[5]Department of Medical Lifescience, College of Medicine, College of Medicine, Catholic University of Korea, 222 Banpo-daero, Seocho-gu, Seoul 06591, Republic of Korea

To whom correspondence should be addressed to:

Mi-La Cho, Ph.D., Rheumatism Research Center, Catholic Research Institute of Medical Science, College of Medicine, The Catholic University of Korea, Seoul 06591, Korea (Tel: 82-2-2258-7473, Fax: 82-2-2258-7473, E-mail: iammila@catholic.ac.kr)

Table 1. List of primers for real-time PCR in this study

Gene Name	Forward Primer	Reverse Primer
**MMP1**	CTGAAGGTGATGAAGCAGCC	AGTCCAAGAGAATGGCCGAG
**MMP3**	CTCACAGACCTGACTCGGTT	CACGCCTGAAGGAAGAGATG
**MMP13**	CTATGGTCCAGGAGATGAAG	AGAGTCTTGCCTGTATCCTC
**TIMP1**	AATTCCGACCTCGTCATCAG	TGCAGTTTTCCAGCAATGAG
**TIMP3**	CTGACAGGTCGCGTCTATGA	GGCGTAGTGTTTGGACTGGT
**NFκB**	AACAGAGAGGATTTCGTTTCCG	GGACTTCGAGCAAGAGATGG
**iNOS**	CCTGAGCTCTTCGAAATCCCA	CCCGAAACCACTCGTATTTGG
**RIPK1**	GACGAAGCCAACTACCATCTT	TCTCCTTTCCTCCTCTCTGTT
**MLKL**	GCAAGCTGGTGGCTGTGAAGC	TTCATCCACAGAGGGCCGCA
**β-actin**	GGACTTCGAGCAAGAGATGG	TGTGTTGGCGTACAGGTCTTTG

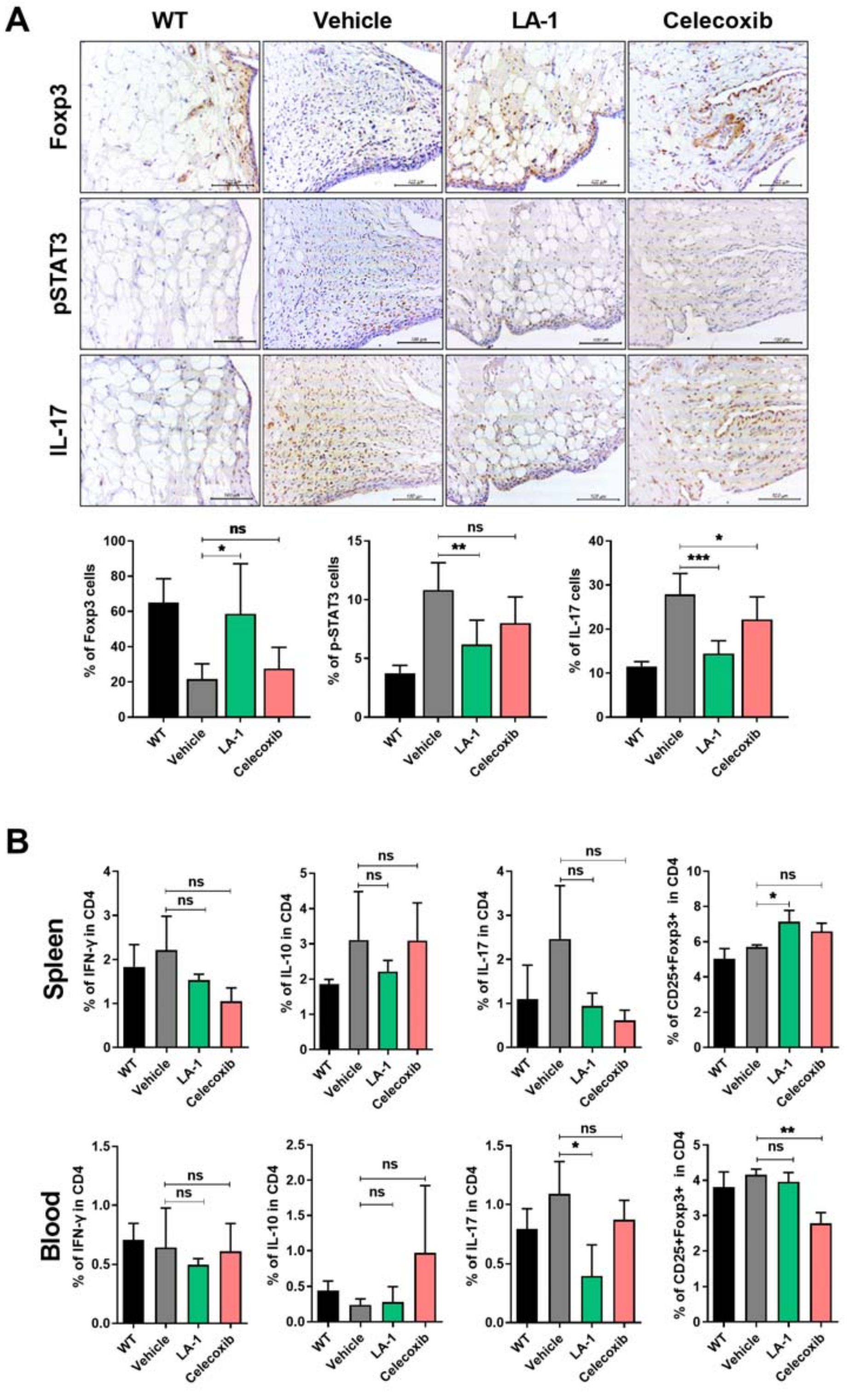

A
WT
Vehicle
LA-1
Celecoxib
Foxp3
pSTAT3
IL-17
% of Foxp3 cells
% of p-STAT3 cells
% of IL-17 cells
ns
*
**
***
B
Spleen
Blood
% of IFN-γ in CD4
% of IL-10 in CD4
% of IL-17 in CD4
% of CD25+Foxp3+ in CD4

Supplementary Figure 1. Lactobacillus regulates the expression of inflammatory and regulatory T cells. (A) FOXP3, p-STAT3, and IL-17 expression determined immunohistochemically in the synovium of indicated group. (B) Bar graphs show average % of IFN-γ, IL-10, and IL-17 positive $CD4^{+}$ T cells of spleen (top) and blood (bottom) of indicated group. (C) Bar graphs show average % of $CD25^{high}FOXP3^{high}CD4^{+}$ T cells (Treg) of spleen (top) and blood (bottom) of indicated group. Data are means ± SD ($*p < 0.05$, $**p < 0.01$, $****p < 0.001$).

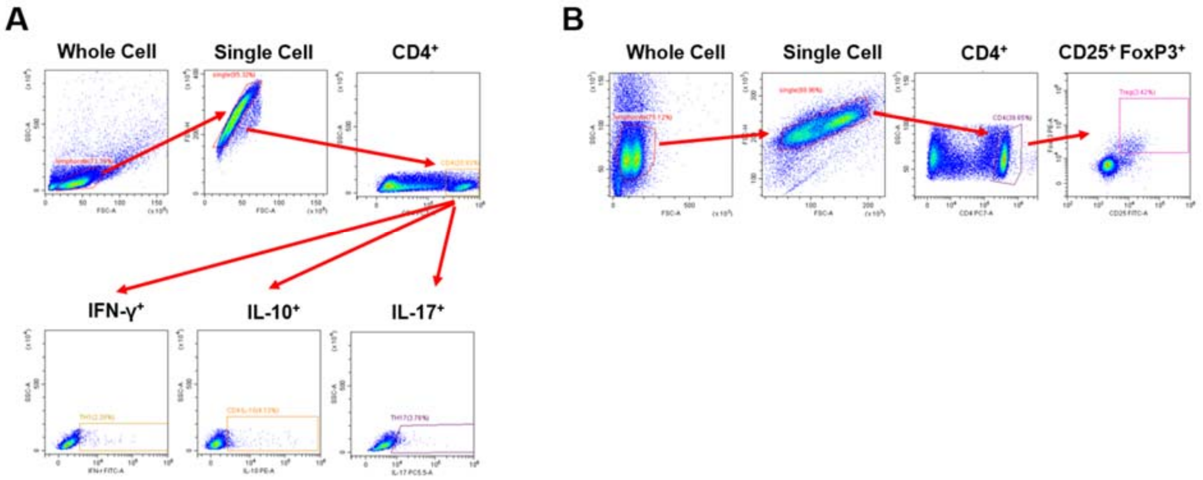

Supplementary Figure 2. An example of gating strategy for flow cytometry. (A) Gating strategy for IFN-γ^{+}, IL-10^{+}, and IL-17^{+} CD4+ T cells. (B) Gating strategy for $CD4^{+}CD25^{+}FoxP3^{+}$ cells (Tregs).

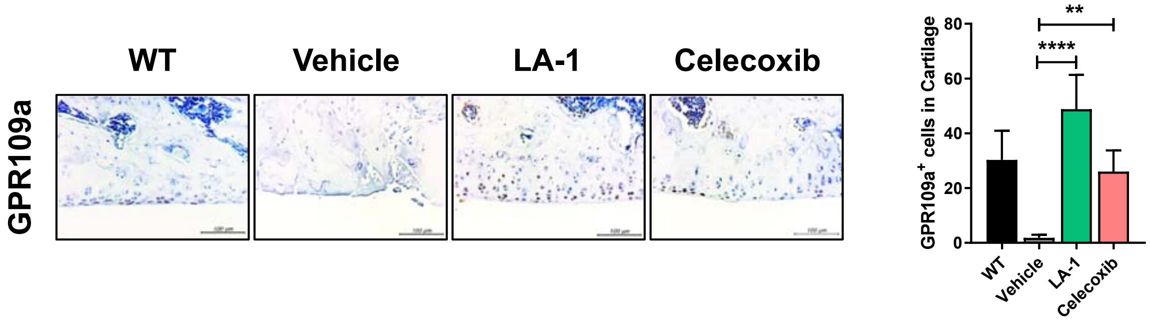

Supplementary Figure 3. Regulation of GPR109a expression by LA-1. Representative images show the expression of GPR109a and bar graph shows GPR109a positive cell number in the indicated group.

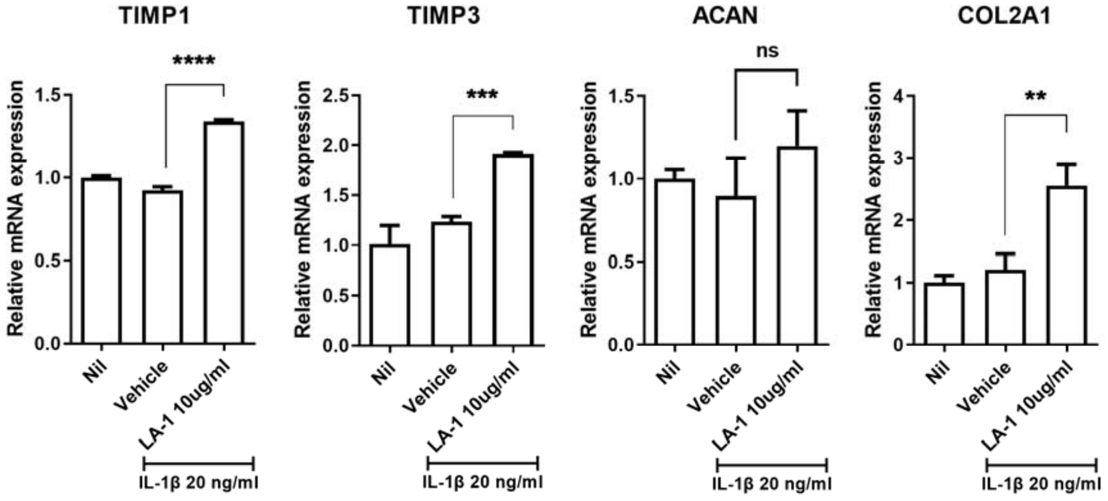

Supplementary Figure 4. Lactobacillus regulate the expression of anabolic factors & ECM - related gene. Bar graphs show the mRNA expression level of TIMP1, TIMP3, ACAN, and COL2A1 in the human chondrocyte of indicated group. Data are means ± SD (**$p < 0.01$, ***$p < 0.005$, ****$p < 0.001$).

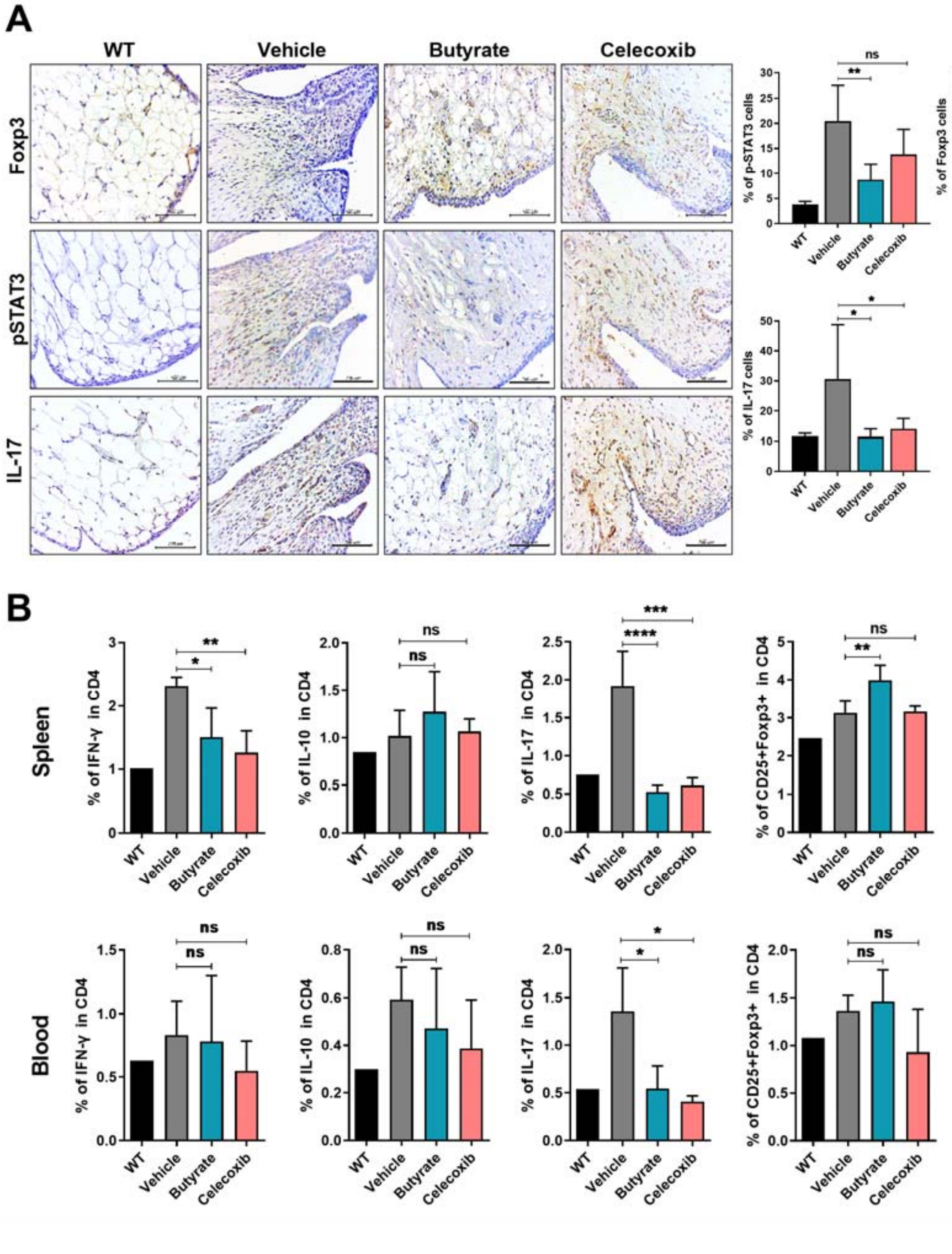
A
WT
Vehicle
Butyrate
Celecoxib
Foxp3
pSTAT3
IL-17
% of p-STAT3 cells
% of Foxp3 cells
% of IL-17 cells
ns
**
*
B
Spleen
Blood
% of IFN-γ in CD4
% of IL-10 in CD4
% of IL-17 in CD4
% of CD25+Foxp3+ in CD4
***
****

Supplementary Figure 5. Butyrate regulates the expression of inflammatory and regulatory T cells. (A) FOXP3, p-STAT3, and IL-17 expression determined immunohistochemically in the synovium of indicated group. (B) Bar graphs show average % of IFN-γ, IL-10, and IL-17 positive $CD4^+$ T cells of spleen (top) and blood (bottom) of indicated group. (C) Bar graphs show average % of $CD25^{high}FOXP3^{high}CD4^+$ T cells (Treg) of spleen (top) and blood (bottom) of indicated group. Data are means ± SD (*$p < 0.05$, **$p < 0.01$).

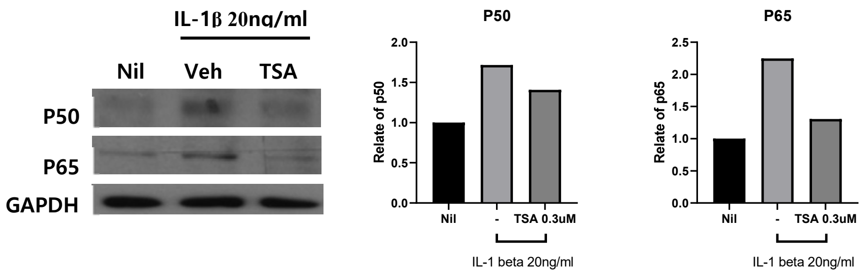

Supplementary Figure 6. Regulatory effect of HDAC inhibitor in the expression of NF-κB. Representative images show the expression of P50 and P65, and bar graphs show quantification of them in the indicated group.

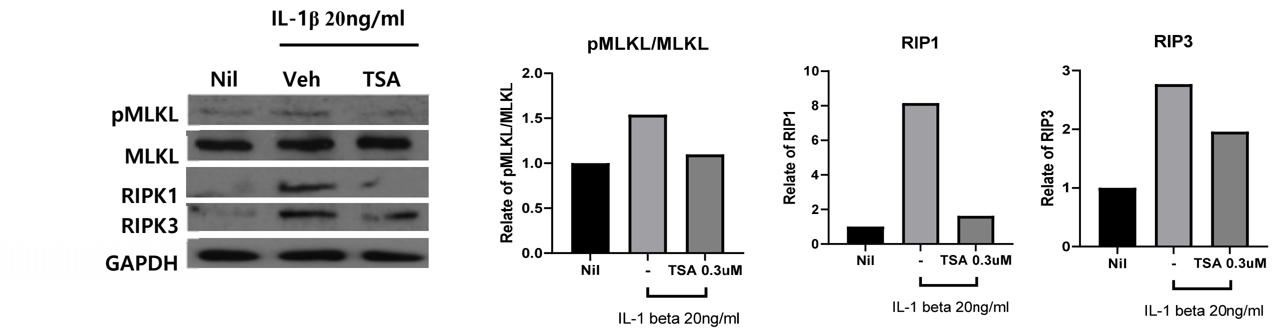

Supplementary Figure 7. HDAC inhibitor inhibit inflammatory cell death. Representative images show the expression of pMLKL, MLKL, RIPK1, and RIPK3, and bar graphs show quantification of them in the indicated group.
